# Good-Quality mHealth Apps for Endometriosis Care: Systematic Search

**DOI:** 10.2196/49654

**Published:** 2025-02-07

**Authors:** Diksha Sirohi, Cecilia HM Ng, Niranjan Bidargaddi, Helen Slater, Melissa Parker, M Louise Hull, Rebecca O'Hara

**Affiliations:** 1 Robinson Research Institute Adelaide Medical School University of Adelaide North Adelaide Australia; 2 School of Clinical Medicine, Medicine and Health UNSW Medicine Sydney Australia; 3 Jean Hailes for Women's Health Melbourne Australia; 4 Digital Psychiatry & Personal Health Informatics Lab College of Medicine and Public Health Flinders University Adelaide Australia; 5 School of Physiotherapy and Exercise Science Faculty of Health Sciences Curtin University Perth Australia; 6 Canberra Endometriosis Centre, Centenary Hospital for Women and Children ACT Health Canberra Australia

**Keywords:** adenomyosis, endometriosis, m-health apps, mobile apps, digital health, pelvic pain, self-learning, clinicians, mHealth, application, endometriomas, chocolate cysts, uterus, womb, pain management, digital health, women's health, mobile phone

## Abstract

**Background:**

Mobile health (mHealth) apps are increasingly being used by community members to track symptoms and manage endometriosis. In addition, clinicians use mHealth apps for continued medical education and clinical decision-making and recommend good-quality apps to patients. However, poor-quality apps can spread misinformation or provide recommendations that are not evidence-based. Therefore, a critical evaluation is needed to assess and recommend good-quality endometriosis mHealth apps.

**Objective:**

This study aimed to evaluate the quality and provide recommendations for good quality endometriosis mHealth apps for the community and clinicians.

**Methods:**

PRISMA (Preferred Reporting Items for Systematic Reviews and Meta-Analyses) 2020 guidelines informed the search of mHealth apps on the Google Play Store and Apple App Store. The search terms included “endometriosis,” “adenomyosis,” and “pelvic pain.” mHealth apps were eligible if they were (1) related to the search terms, (2) were in the English language, and (3) were available free of cost. Only the free content of the eligible mHealth apps was assessed. ENLIGHT, a validated evaluation tool for mobile and web-based interventions, was used to assess the quality across 7 domains such as usability, visual design, user engagement, content, therapeutic persuasiveness, therapeutic alliance, and general subjective evaluation. mHealth apps with a total score of ≥3.5 were classified as “good” according to the ENLIGHT scoring system and are recommended as good-quality mHealth apps for endometriosis care.

**Results:**

In total, 42 mHealth apps were screened, and 19 were included in the quality assessment. A total of 6 good-quality mHealth apps were identified (QENDO, Bearable, Luna for Health, Matilda Health, Branch Health: Pain Management, and CHARLI Health). These apps provided symptom-tracking functions and self-management support. A total of 17 apps were designed for community use, while 2 apps provided a digital endometriosis classification tool to clinicians. Most mHealth apps scored well (≥3.5) in the domains of usability (16/19, 84.2%), visual design (14/19, 73.7%), user engagement (11/19, 57.9%), and content (15/19, 78.9%). Few eHealth websites scored well on therapeutic persuasiveness (6/19, 31.6%), therapeutic alliance (9/19, 47.4%), and general subjective evaluation (6/19, 31.6%).

**Conclusions:**

Although time and geographical location can influence the search results, we identified 6 “good-quality” endometriosis mHealth apps that can be recommended to the endometriosis community. mHealth apps designed for community use should evaluate their effectiveness on user’s endometriosis knowledge, self-recommended management strategies, pain self-efficacy, user satisfaction, and user quality of life. Digital technology should be leveraged to develop mHealth apps for clinicians that contribute to continued medical education and assist clinical decision-making in endometriosis management. Factors that enhance usability, visual design, therapeutic persuasiveness, and therapeutic alliance should be incorporated to ensure successful and long-term uptake of mHealth apps.

**Trial Registration:**

PROSPERO CRD42020185475; https://tinyurl.com/384dkkmj

## Introduction

Endometriosis is a chronic condition characterized by the growth of endometrial-like tissue outside the uterus, causing pain and fertility problems in 190 million women and those assigned female at birth globally [1]. Associated with delayed diagnosis and requiring long-term, individualized care, access to health resources and services is critical to overcoming the negative impact of endometriosis on quality of life and overall well-being [2]. To address this community need, an increasing number of endometriosis mobile health (mHealth) apps [3] have been developed to provide rapid mobile access to symptom assessment and tracking, education, appointment management, and support networking [4]. The value of these mHealth resources in endometriosis care became particularly apparent during COVID-19 lockdowns, when access to doctors and surgery was limited, and elevated endometriosis-related symptoms were observed [5].

As patients increasingly seek digital health information and education [6], medical consultations are progressively more likely to reference mHealth apps. Joint sharing of digital information improves communication between patients and clinicians and enables people to become partners in their health care [4,7,8]. Advancements in digital technologies and the application of machine learning algorithms have led to the development of mHealth interventions [9] to provide more personalized health management [10]. Ball et al [11] trialed a mHealth app intervention to assess the effectiveness of a mindfulness program on chronic pelvic pain in women [11]. In other chronic conditions, including type 1 and type 2 diabetes and hypertension, the integration of digital health strategies led to demonstrable improvements in treatment compliance, disease outcomes [3,11,12], and self-management [13-15].

Clinicians benefit by being informed of the quality and function of endometriosis mHealth apps, as they can provide recommendations to endometriosis community members in a time-efficient, evidenced-based way. There is evidence that mHealth apps can facilitate the diagnosis of endometriosis [16]. The Nezhat Endometriosis Advisor app was trialed to screen populations for the risk of endometriosis, which can assist in early diagnosis [16]. mHealth apps are effective tools that significantly improve medical knowledge and skills among health care providers [17]. The majority of doctors (86%, 170/198) working in pediatric emergency departments across the United Kingdom and Ireland reported using mHealth apps on their phones for clinical decision-making [18]. Ease of use and rapid accessibility facilitates mHealth app use among clinicians [18].

However, poor-quality mHealth apps can spread misinformation [4] and create mistrust in clinical relationships. Furthermore, it is difficult to assess the quality of endometriosis mHealth apps in app stores. There is a need to systematically evaluate endometriosis mHealth apps to inform the endometriosis community and clinicians about the advantages and disadvantages of those currently available using validated and standardized tools [9,19]. Therefore, the aim of this study was to conduct a systematic search of mHealth apps that support endometriosis care to evaluate the quality and recommend those apps that meet specific quality metrics to the endometriosis community and clinicians.

## Methods

### Search Strategy

This systematic search was conducted in accordance with the PRISMA (Preferred Reporting Items for Systematic Reviews and Meta-Analysis) guidelines [20]. (Multimedia Appendix 1). Before undertaking this study, a systematic search protocol was developed and registered with the International Prospective Register of Systematic Reviews (PROSPERO; registration CRD42020185475). There was no patient or public involvement in this study.

“Endometriosis,” “adenomyosis,” and “pelvic pain” were used as search terms in the 2 largest app stores (Google Play and Apple) on July 24, 2020. Only the first 30 listed mHealth apps were screened, as there is evidence that most people do not investigate beyond this number [21]. Each term was separately searched in the Apple app and Google Play store. The search was later updated on July 22, 2024, to include new mHealth apps and remove redundant ones. Duplicate results were removed, and the remaining mHealth apps were screened for eligibility.

#### Inclusion and Exclusion Criteria

mHealth apps were included if they related to endometriosis, adenomyosis, or pelvic pain, were in the English language, and were available free of cost. mHealth apps that did not meet the inclusion criteria were excluded (Textbox 1). There were no geographical limitations. For those mHealth apps with free and paid membership sections, only the free components were assessed. In-app purchases were not made.

**Inclusion and exclusion criteria for including mobile health (mHealth) apps for endometriosis care.**

**Inclusion criteria:**

- Relevance to the subject: mHealth apps that relate to endometriosis, adenomyosis, or pelvic pain in women.
- Cost: Free mHealth apps (no associated cost) or free components of mHealth apps.
- Language: mHealth apps written in the English language.

**Exclusion criteria:**

- Relevance to the subject: mHealth apps that did not relate to endometriosis, adenomyosis, or pelvic pain in women.
- Cost: mHealth apps that require a payment or subscription to access them.
- Language: mHealth apps written in a language other than English.

#### Data Extraction

Descriptive data was manually extracted by 1 researcher (DS) after reading an explanation of each mHealth app’s purpose. This data was collated in a Microsoft Excel spreadsheet under the following categories: (1) mHealth app name, (2) hyperlink, (3) developer, (4) version, (5) stated purpose, (6) target audience, (7) category, (8) country of origin, (9) last updated, and (10) user rating (Multimedia Appendix 2). The target audience was classified into 2 categories: (1) the endometriosis community (eg, laypeople in the community who have endometriosis or pelvic pain) and (2) clinicians. mHealth apps were classified into 5 types: (1) symptom trackers, which allowed users to record and analyze their symptoms; (2) endometriosis screening tools, which assessed the probability of having endometriosis; (3) pelvic pain symptom assessment tools, which analyzed users pelvic pain symptoms to suggest self-management strategies; (4) a fertility coaching program for those affected with endometriosis; and (5) endometriosis classification tools, which assisted clinicians with endometriosis classification based on radiological and/or surgical findings.

#### Quality Assessment

The ENLIGHT quality assessment tool was used to evaluate all included mHealth apps [22]. The ENLIGHT tool assesses 7 criteria: (1) usability, (2) visual design, (3) user engagement, (4) content, (5) therapeutic persuasiveness, (6) therapeutic alliance, and (7) general subjective evaluation (Table 1).

**Table 1:** Description of the ENLIGHT quality assessment criteria [22], objectives, and factors assessed.

Domains of quality assessment	Objective	Criteria assessed
Usability	Assesses the ease of learning how to use a mHealth app and the ease of using it appropriately.	Navigation Learnability Ease of use
Visual design	Assesses the look and feel of the mHealth app and the visual quality of the Graphical User Interface (GUI).	Aesthetics Layout Size
User engagement	Assesses the extent to which the mHealth app design attracts users to use it.	Content presentation Interactive Not irritating Targeted/tailored/personalized reports Captivating
Content	Assesses the content provided or learned while using the mHealth app.	Evidence-based content Quality of information provided Complete and concise Clarity about the program’s purpose
Therapeutic persuasiveness	Assesses the extent to which the mHealth app is designed to encourage users to make positive behavior changes or to maintain positive aspects of their lives.	Call to action Load reduction of activities Therapeutic rationale and pathway Rewards Real data-driven/adaptive content On-going feedback Expectations and relevance
Therapeutic alliance	Assesses the ability of the mHealth app to create an alliance with the user to effect a beneficial change.	Basic acceptance and support Positive therapeutic expectations Relatability
General subjective evaluation of program’s potential	Examines the mHealth app’s general potential to benefit its target audience based on the rater’s subjective evaluation	Appropriate features to meet the clinical aim Right mix of ability and motivation I like the program

The mHealth apps were reviewed in 2 stages. Initially, one researcher (DS) reviewed all included mHealth apps. Then, the mHealth apps were divided and reviewed by a team of six independent researchers (RO, MLH, NB, HS, MP, and CHMN). Hence, each app was reviewed by 2 researchers (DS and 1 of the 6 researchers).

All reviewers were required to download the mHealth app to their smartphone devices (mobile phone or tablet). The ENLIGHT scores of each mHealth app were collated in an Excel spreadsheet. Each ENLIGHT quality assessment criteria was scored using a rating scale of 1-5 (Very Poor to Very Good) using the ENLIGHT checklist [22]

Discrepancies in ratings (any deviation greater than 1 rating unit) were resolved by discussion between the two reviewers. If evaluation differences were not resolved, a third independent assessor was consulted. After a detailed assessment, the average of the 2 reviewer’s ratings was used to calculate a score for each of the 7 domains.

#### Good-Quality mHealth Apps

A total score for each mHealth app was calculated according to the ENLIGHT formula (Multimedia Appendix 3). mHealth apps with a total score of ≥3.5 were classified as “good” according to the ENLIGHT scoring system [23] and were recommended as good-quality mHealth apps for endometriosis care.

### Ethical Considerations

An ethics approval was not required for this study, because this was a systematic search of mHealth apps and did not involve the recruitment of participants.

## Results

**Figure 1:**
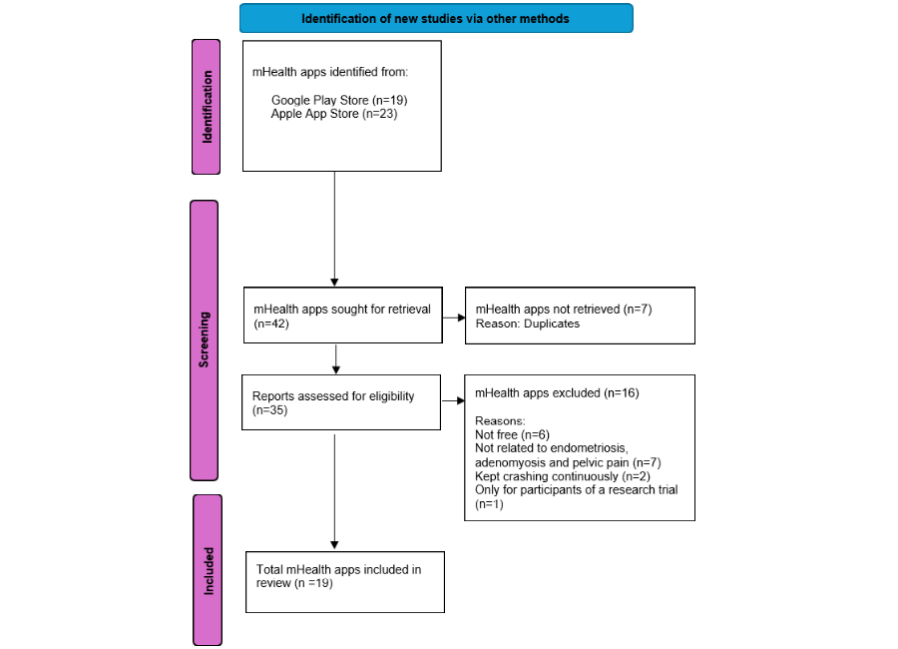
Flow chart showing the selection of mobile health (mHealth) apps from Apple App and Google Play stores. Adapted from PRISMA (Preferred Reporting Items for Systematic Reviews and Meta-Analyses) 2020 flow diagram.

The mHealth app search identified 42 apps, 23 from the Apple App Store and 19 from the Google Play store. A total of 7 mHealth apps were duplicates, leaving 35 apps, of which 13 were excluded as they did not meet the inclusion criteria (Figure 1). A further 3 mHealth apps were excluded; of these, 2 were excluded due to technical difficulties with the app (eg, it kept crashing), and one was excluded as it was specifically for participants enrolled in a research study. A total of 19 mHealth apps were included in the final analysis (Multimedia Appendix 2). There were discrepancies between the first and second reviewers in 6% of the ratings. All discrepancies were resolved without needing a third reviewer through consensus meetings.

Out of the 19 included mHealth apps, the majority (n=16) were designed for use by laypeople in the community. In addition, 2 mHealth apps were designed for health care providers. The majority of apps mainly provided symptom tracking functions (73.7%, n=14), 2 apps provided digital endometriosis classification tools (10.5%), 1 app offered an endometriosis fertility-related coaching program (5.3%), 1 app provided an endometriosis screening tool (5.3%), and 1 app offered a pelvic pain assessment tool (5.3%).

### Quality of mHealth Apps

A total of 6 mHealth apps met the cutoff criteria for classification as “good” (ie, final score >3.5 to 5) according to the ENLIGHT quality assessment criteria. (Table 2). These mHealth apps are recommended as “good quality” apps for endometriosis care for community use.

**Table 2:** “Good” scoring (≥3.5 to 5) mHealth apps using the ENLIGHT Quality Assessment criteria (based on the final score).

Name	Usability	Visual design	User engagement	Content	TP^a^	TA^b^	GSE^c^	Final score
QENDO [24]	4.67	4.50	4.80	4.38	4.21	4.33	4.00	4.36
Bearable [25]	4.50	4.50	4.40	4.00	4.29	4.00	4.50	4.23
Luna for Health [26]	4.17	4.67	4.10	3.88	4.14	3.50	3.50	4.03
Matilda Health [27]	5.00	4.83	4.60	4.00	3.64	4.50	4.17	3.96
Branch Health: Pain Management [28]	4.00	4.17	3.70	3.50	3.71	3.67	2.83	3.67
CHARLI Health [29]	4.00	3.33	3.40	4.13	3.36	3.50	2.83	3.52

^a^TP: therapeutic persuasiveness.

^b^TA: therapeutic alliance.

^c^GSE: general subjective evaluation.

### Usability

Approximately 84.2% (16/19) of the mHealth apps scored “good” (≥3.5) on usability (Multimedia Appendix 3). The highest-ranking mHealth apps were characterized by smooth, nearly frictionless, and easy navigation. These apps also had an intuitive interface that facilitated ease of learning and straightforward use of the app. Their structure was self-explanatory, and the user required minimum effort to obtain the desired output. Some examples of mHealth apps that scored “good" under this domain include QENDO [24], Bearable [25], Matilda Health [26], #Enzian [30], and the American Association of Gynecologic Laparoscopists (AAGL) Endo Classification app (Medicinia Atividades de Internet Ltda) [31]. While the first 3 mHealth apps are designed for community use, the last 2 can be used as resources in clinical practice.

### Visual Design

The majority of mHealth apps (73.7%, 14/19) scored “good” (>3.5) on visual design. These mHealth apps (1) had an overall attractive visual design and an appealing color scheme (eg, QENDO [24]), (2) were well structured with a consistent layout (eg, Branch Health Pain Management (Upside Health) [28]), (3) clearly presented content that was easy to read (eg, Luna for Health; HDSI) [26]), and (4) displayed appropriately sized fonts, buttons, and menus (eg, Frendo [32]).

### User Engagement

More than half of the mHealth apps scored ≥3.5 on user engagement (57.9%, 11/19). These were characterized by a good mix of text, images, and videos. The content was presented in an interactive and engaging manner. User engagement was further enhanced by avoiding features like pop-up ads, notifications, alerts, and sounds. Some mHealth apps in this domain provided tailored, targeted, and personalized user-specific health reports (eg, QENDO [24] and Matilda Health [27]) and/or charts (eg, Bearable [25]) that users could take to their health appointments. Users of the Luna for Health app [26], in addition to symptom tracking, could also undertake a menstrual well-being test that suggested the need for further medical evaluation.

### Content

The majority of mHealth apps scored ≥3.5 under content (78.9%, 15/19). These mHealth apps contained appropriate, complete, and concise information with good clarity about the app’s purpose. Some examples include QENDO [24], #Enzian [30], and Flutter [33]. Amongst the 15 apps that scored well in this domain, only 1 app was designed to assist clinicians (#Enzian [30]) in the classification of endometriosis based on radiological and/or surgical findings.

### Therapeutic Persuasiveness

A low number of mHealth apps (31.6%, 6/19) scored ≥3.5 under the therapeutic persuasiveness. These apps had a clear call to action and engaged the user by developing health plans or health reports with an actionable outcome. For example, the Bearable app [25] allowed users to set goals like exercising, limiting caffeine intake, or avoiding screen time 1 hour before bedtime. Straightforward and easy-to-complete activities in these apps included completing a health assessment questionnaire, data entry for symptom tracking, and documenting daily challenges. For example, QENDO [24] provided users with a report to take to (or email) their doctor. Matilda Health [27] allowed users to schedule webinars of interest or ask questions to clinicians. The aim of these activities was clear to users. The “good” scoring apps also provided ongoing feedback about the user’s health or recommendations regarding testing and likely diagnosis based on symptoms.

### Therapeutic Alliance

Less than half (47.4%, 9/19) of the mHealth apps scored “good”(≥3.5) under the therapeutic alliance. These mHealth apps demonstrated basic acceptance, support, and relatability in creative ways. Features included (1) the use of supportive language (for example, the Luna for Health app [26] used phrases like “you are not alone in this journey”), (2) positive therapeutic support (eg, CHARLI Health; Lucid Labs Pty Ltd) [29] provided an option to chat with doctors and endometriosis specialists) and (3) an online community that fosters a sense of connectedness (eg, Flutter [33]).

### General Evaluation of the mHealth Apps

This criterion evaluates the reviewers’ subjective global score. A low number of mHealth apps (31.6%, 6/19) scored “good” (≥3.5) in this domain. The reviewers felt that they displayed features appropriate to their aims, had the elements to motivate the use of the app, and were generally likable. Some examples include QENDO [24] and Matilda Health [27].

## Discussion

### Principal Findings

We conducted a comprehensive, multidimensional quality assessment of endometriosis mHealth apps using the ENLIGHT tool [22]. This tool captures quality constructs like therapeutic persuasive design and therapeutic alliance, which are considered central to the successful uptake of mHealth apps among end users. Our study identified six “good” quality endometriosis mHealth apps that can be recommended for consideration in supporting the community in endometriosis care. Additionally, two mHealth apps can be recommended for consideration in assisting healthcare providers in the classification of endometriosis. To our knowledge, this is the first systematic search and assessment to use the ENLIGHT tool to comprehensively assess endometriosis and pelvic pain mHealth apps.

### Comparison With Previous Work

#### mHealth Apps for the Endometriosis Community

In total, 17 out of the 19 mHealth apps in this study could be used by people in the community. mHealth apps are widely used for symptom tracking and self-management of menstrual pain [9]. We found that most mHealth apps were designed for community use, facilitated symptom tracking, and provided users with symptoms and/or activity reports that could be shared with health care providers. Such tracking and activity reporting appear to be desirable features of mHealth apps [34]. A study reported that 77.7% of people affected with endometriosis were comfortable sharing data tracked on mHealth apps with clinicians, indicating the acceptability of data sharing for health care management [34]. The Bearable app [25] provides users with a correlations grid, demonstrating the impact of certain activities like meditation, exercise, or diet on symptoms, mood, sleep, and/or energy levels. Similarly, the Phendo app (Citizen Endo) [35] provided tailored insights on self-management strategies. In addition, Phendo [35] is a research app, which has used direct self-reported data to study the heterogeneity of endometriosis symptoms [36]. This can help analyze symptom variability among people with endometriosis [36,37], demonstrating the usefulness of digitally generated data in endometriosis research. Self-tracked data from mHealth apps can be used to analyze the association between symptoms and periodicity as potential health indicators for endometriosis assessment [37]. Furthermore, such data can be used as an additional data source that complements patient electronic health records to accurately and comprehensively evaluate patient health history [36].

Apps like Luna for Health [26], Matilda Health [27], and CHARLI Health [29] offer virtual consultations with clinicians specialized in endometriosis and with allied health professionals like physiotherapists and dietitians providing the community with comprehensive alternate pathways for endometriosis care through digital technology. These digital advancements build capacity to meet the gap in the health workforce and improve accessibility for endometriosis management. Data tracking, real-time visual representation of symptoms, the effectiveness of treatments, and self-management strategies can assist clinicians in planning more personalized endometriosis treatments in the future [34].

The effectiveness of mHealth apps for other chronic diseases has previously been evaluated. For example, a Canadian mHealth app, “bant” (University Health Network, Toronto, Ontario), developed for adolescents to manage type 1 diabetes, increased the daily average monitoring of blood glucose levels and demonstrated high user satisfaction [38]. Kollmann et al [39] found a significant decrease in hemoglobin A_1c_ levels using the Diab-Memory App in patients with type 1 diabetes. A mobile phone–based technology called NICHE [12] achieved similar results in patients with type 2 diabetes. There appears to be limited evidence on the evaluation of endometriosis mHealth apps. The Endo-App (Endo Health GmBH) [40] evaluated the impact of the app on disease-related quality of life and symptoms of endometriosis in 122 participants using a randomized controlled pilot trial [41]. Participants in the intervention group reported clinically relevant and statistically significant improvements in pain-specific self-efficacy, fatigue, depression, and quality of life after 12 weeks of use as compared with controls [41], demonstrating the scope of mHealth apps to improve the well-being of those affected by endometriosis. Given this potential, mHealth apps should ideally evaluate their effectiveness on users’ quality of life, impact on endometriosis knowledge, the effectiveness of recommended self-management strategies, and user satisfaction or outcomes of virtual consultations (such as reduced delay in diagnosis or impact on consultation wait times).

#### Endometriosis mHealth Apps as a Resource in Clinical Practice

This study found only 2 mHealth apps designed specifically for health care providers, both of which offered digital tools to classify endometriosis based on radiological and/or surgical findings. We did not find any mHealth app that provided education to clinicians, yet the need to improve endometriosis awareness among clinicians is expressed by both, the community [2,42] and clinicians [43]. A Dutch study reported that 87% (76/87) of general practitioners (GPs) reported the need to improve endometriosis education with the same study reporting that GPs scored 40% on endometriosis factual knowledge (ie, questions regarding pathophysiology) and 49.4% on clinical knowledge (ie, questions regarding endometriosis symptoms and treatment) [43].

Furthermore, mHealth apps are increasingly being used by clinicians for medical education, training, and clinical decision-making [44]. A study showed that 98.4% of doctors own a smartphone and 92% agree that mHealth apps positively impact clinical practice [45]. In this context, a meta-analysis reported that the pooled effect of 15 studies with 962 participants showed significant improvement in knowledge scores among health care providers who used mHealth apps compared with those who did not use them [17]. Previously, the Endo App by the European Society for Human Reproduction and Embryology (ESHRE) [46] provided a clinical decision-making tool to clinicians in busy clinical settings. Through the decision tool, clinicians could also conveniently access the ESHRE endometriosis clinical practice guidelines [47], which were time-saving in their daily practice. However, this app is no longer available through the app store and appears to have been discontinued. Given the rising popularity and uptake of mHealth apps among clinicians and the need for endometriosis education, digital technology can be leveraged to provide endometriosis-related continuing medical education and clinical decision-making tools for clinicians.

#### The ENLIGHT Quality Assessment

The ENLIGHT criteria evaluated factors that are central to the long-term and successful uptake of the mHealth apps. The majority of the mHealth apps scored “good” (≥3.5) on usability (84.2%) and visual design (73.7%). However, only 31.6% and 47.4% of mHealth apps scored “good” on user therapeutic persuasiveness and therapeutic alliance respectively. Usability and visual design are important features that determine the success and user uptake of mHealth apps [48,49]. mHealth apps that provide personalized and tailored feedback have demonstrated better user engagement [50,51]. A mental health, microrandomized, clinical trial reported that using push notifications to send a tailored health message was an effective strategy to increase user engagement [50]. Therapeutic persuasiveness is positively correlated with real-world usage of mHealth apps and is an important predictor of user adherence [52]. The ENLIGHT criteria assess the use of rewards to increase therapeutic persuasiveness [21]. Rewards are effective at motivating people [53]. One mHealth app intervention successfully used tangible rewards such as redeeming reward points for purchasing iTunes in an adolescent population [38]. Another study found that participants preferred tangible rewards such as gift cards or monetary incentives as compared with intangible rewards like points or badges [15]. We found a few mHealth apps that provided rewards to users. Users of the Luna for Health app [26] earned “Luna Coins” by participating in quizzes; however, it was unclear how these coins could be used. The therapeutic alliance enhances positive user engagement by fostering relatability [21,52]. It may be that future apps can incorporate conversational agents such as smartphone-based chatbots portraying human involvement [52,54,55] or conversational agents such as Alexa (Amazon), which mimic human conversations [54]. Although mHealth apps are not human, the extent to which they build an alliance is integral to their quality [21].

#### Strengths

Our systematic search and assessment of the quality of mHealth apps for endometriosis has several strengths. First, it presents novel findings on “good” quality mHealth apps developed for endometriosis care and relevant to the community. We identified mHealth apps that could be considered as a potentially useful resource for use in clinical practice. Clinicians may use the findings of this study to consider recommendations on mHealth app use to support people and families affected by endometriosis. Finally, our study highlights features of therapeutic persuasiveness and therapeutic alliance and additional quality metrics that can be incorporated into future developments of mHealth apps for endometriosis.

### Limitations

The ENLIGHT criteria [20] were challenging to uniformly apply. The evaluation of evidence-based content was challenging as most mHealth apps were symptom trackers and presented limited evidence-related content. The quality of content was assessed based on material available in the free version of the app. However, most apps contained endometriosis content and learning modules as part of their paid version. For example, with both, Luna for Health [26] and Matilda Health [27], we were unable to evaluate content completely as we were only able to assess freely available content. In the #Enzian [30] and AAGL Endo Classification app [31], “rewards” and “ongoing feedback,” which form part of therapeutic persuasiveness were not features offered to users. Hence, therapeutic persuasiveness was difficult to evaluate, with limited formal guidance on how to navigate such scenarios. Furthermore, this study did not include non-English mHealth apps, due to limitations in funding for translation services. In addition, due to the search algorithm on both app stores that used geographical location, that is, Australia, which is predominantly an English-speaking country, we did not come across any app that was not developed in the English language.

### Conclusions

Our systematic search presents novel findings on the quality assessment of mHealth apps that can be used for endometriosis care. Based on the use of a validated tool for rating app quality metrics, our findings are suggestive of “good quality” mHealth apps for community use. These quality ratings can be considered by clinicians as potential recommendations to patients to support their care. Clinicians can use 2 mHealth apps for endometriosis classification in clinical practice. To further strengthen app development for the endometriosis community, we recommend that (1) endometriosis-related mHealth apps incorporate the standardized experience and outcome measures to assess the effectiveness of programs offered. For example, user satisfaction, effectiveness of recommended self-management strategies, impact on user’s endometriosis knowledge, pain self-efficacy, and overall quality of life, (2) digital technology is leveraged to develop clinical resources for endometriosis-related continued medical education and to support clinical decision-making, and (3) good design features are considered more carefully including therapeutic persuasiveness and therapeutic alliance for the successful uptake of endometriosis mHealth apps.

### Authors’ Expertise in Conducting the Search and Quality Assessment

The interprofessional team consisted of endometriosis researchers, clinical pain researchers, clinicians (gynecologist, specialist physiotherapist, and endometriosis nurse specialist), and digital health experts. The team has expertise in the clinical management of endometriosis, community engagement, co-design of digital health tools, endometriosis content, and evaluation research to support self-management and care. All authors were involved in the quality assessment.

## Appendix

Multimedia Appendix 1 PRISMA (Preferred Reporting Items for Systematic Reviews and Meta-Analyses) 2020 Checklist and PRISMA 2020 Abstracts Checklist.

Multimedia Appendix 2 Details of mobile health apps included in this study.

Multimedia Appendix 3 Final score of mobile health apps based on the ENLIGHT scoring system.

## Abbreviations

AAGL: American Association of Gynaecologic Laparoscopists
ESHRE: European Society for Human Reproduction and Embryology
GP: general practitioner
mHealth: mobile health
PRISMA: Preferred Reporting Items for Systematic Reviews and Meta-Analyses
PROSPERO: International Prospective Register of Systematic Reviews

## Data Availability

All data generated or analyzed during this study are included in this published article and Multimedia Appendices 1-3.
